# Frequency and Clinical Features of Space Headache Experienced by Astronauts During Long-Haul Space Flights

**DOI:** 10.1212/WNL.0000000000209224

**Published:** 2024-03-13

**Authors:** Willebrordus P.J. van Oosterhout, Matthijs J.L. Perenboom, Gisela M. Terwindt, Michel D. Ferrari, Alla A. Vein

**Affiliations:** From the Department of Neurology (W.P.J.v.O., M.J.L.P., G.M.T., M.D.F., A.A.V.), Leiden University Medical Center; and Department of Neurology (W.P.J.v.O.), Zaans Medical Center, Zaandam, the Netherlands.

## Abstract

**Background and Objectives:**

Few anecdotal cases and 1 small retrospective study during short-duration space missions suggest that headache may occur early in flight, as part of the space motion syndrome. Whether headaches may also occur at later stages of space flights is unknown. We aimed to prospectively characterize the incidence, timing, clinical features, and management of space headaches during long-duration flights.

**Methods:**

We prospectively evaluated the occurrence, characteristics, and evolution of space headaches and the effects of treatment and countermeasures during long-haul flights with onboard questionnaires and correlated them with prevailing temperature, pressure, and ambient O_2_ and CO_2_ levels, measured within the International Space Station. In addition, we analyzed retrospective headache data from a different astronaut cohort. Headache data were reported using descriptive statistics and correlation data with intraindividual logistic regression models. Astronauts were included through (inter)national aerospace organizations.

**Results:**

In the prospective study, 22/24 (91.7%) astronauts (mean ± SD age: 46.6 ± 6.5 years, 95.8% male) experienced ≥1 episode of headache during a total of 3,596 space days. A total of 378 episodes were reported (median 9; range 1–128) with detailed information on 189. Phenotypically, 170/189 (89.9%) episodes were tension-type headache (TTH) and 19/189 (10.1%) were migraine. Episodes in the first week differed from those in later periods in terms of phenotype (migraine 12/51 [23.5%] vs 7/138 [5.1%]; TTH 39/51 [86.5%] vs 131/138 [94.9%]; overall *p* = 0.0002) and accompanying symptoms: nausea: 17.6% vs 6.9%, *p* = 0.05; vomiting: 9.8% vs 0.7%, *p* = 0.005; nasal congestion: 52.9% vs 29.7%, *p* = 0.004; facial edema: 41.2% vs 1.4%, *p* < 0.001; and duration (*p* = 0.001). Severity and treatments were similar: acute antiheadache medication: 55.6%; other medication: 22.4%; and alternative treatments: 41.1%. Headache occurrence was not associated with temperature or ambient pressure/levels of O_2_ and CO_2_ (all *p* > 0.05). In the retrospective study, 23/42 (54.8%) astronauts (43.5 ± 7.2 years, 90.5% male) reported experiencing ≥1 headache episode during mission. Nasal congestion was the most common (8/33; 24.2%) accompanying symptom. Seventeen of 42 astronauts have been previously described.

**Discussion:**

Astronauts during space flights frequently experience headaches. These most often have characteristics of TTHs but sometimes have migrainous features, particularly during the first week of flight in astronauts without a history of recurrent headaches before or after the space flight.

## Introduction

Space flight–induced microgravity affects the function of multiple body systems, including the brain.^1,2^ In retrospective studies, approximately 70% of astronauts who did not experience recurrent headaches on the Earth reported headaches, including migraines, during short-term space missions.^3,4^ Space headache has now been included in the International Classification of Headache Disorders, third edition (ICHD-3) classification as a secondary headache.^5^ It mainly occurs during the first few days of a space flight and is possibly related to space motion sickness.^6,7^ Anecdotal reports suggest that space headache may also occur at later stages of prolonged space flight, possibly because of prolonged microgravity.^1^ Data on whether headaches do occur at later stages of space flights are lacking.

We wanted to (1) better study whether the symptomatology and occurrence of space headache changed over the course of a space flight, (2) study whether this might be due to prolonged microgravity or other possible mechanisms, and (3) study what therapy is effective in treating space headache. To this extent, we investigated a cohort of astronauts during the early and late phases of long-term space flights aboard a space station. Data were retrospectively collected initially and then prospectively confirmed in a different group. Studying space headaches in otherwise “super healthy” people without a history of recurrent headaches on the Earth might enhance the understanding of headache in general and migraine particularly.

## Methods

The prospective and retrospective data of the study were collected and analyzed separately. To this extent, we investigated a uniquely large cohort of 66 astronauts during the early and late phases of long-term space flights aboard a space station. We prospectively collected data from 24 astronauts and confirmed it with retrospective data from 42 astronauts.

### Headache Questionnaires (Both Studies)

Questionnaires that were developed were based on the criteria of the International Classification of Headache Disorders, 2nd (ICHD-2)^8^ and 3rd (ICHD-3) editions.^5^ In accordance to the ICHD-2 and ICHD-3, the term “stress” was not specified in the question “whether headache was aggravated by stress.”

### Prospective Study

#### Participants

All astronauts from the European Space Agency (ESA), National Aeronautics and Space Administration (NASA), and Japan Aerospace Exploration Agency (JAXA) that were assigned to International Space Station (ISS) expeditions from November 2011 to June 2018 were eligible to participate. We aimed at including long-term data from 24 astronauts.

#### Design

In each astronaut, baseline preflight data were collected 2–6 months before the launch. Astronauts had to complete a questionnaire about their individual headache history before the flight. The preflight questionnaires were coded and anonymized before analysis. Data during the flight were collected using 2 specific types of questionnaires: a daily questionnaire for the first 7 days of the space flight and a weekly questionnaire to be completed each following week throughout the stay in the ISS. The questionnaires were provided digitally through the data system on board the Soyuz vehicle and the ISS, daily for the first week and then weekly until the end of the mission. When more than 1 headache episode occurred per day (in week 1) or per week (after week 1), crew members were required to describe the most severe episode in the questionnaire. ESA, NASA, and JAXA employees and contractors provided and deployed the data software system on the ISS and scheduled all briefings, data collection intervals, and debriefings about the calendars and timelines of the crew members. A semistructured telephone debriefing interview was conducted within 3 months of the flight's return to clarify any unclearly reported details (A.A.V.; W.P.J.v.O.).

#### Data Transfer and Storage

In-flight data were downlinked in encrypted form and provided in encrypted and password-protected files by ESA User Operations Center. Both hard copy preflight and digital in-flight data were sent from the NASA/ESA to the investigators through the Danish Aerospace Company. Data were then stored in the password-protected server of the Department of Neurology of Leiden University Medical Center (LUMC), where only the study team had access to. LUMC's systems are designed for sensitive data and follow all data guidelines.

### Retrospective Study

#### Study Design, Participation, and Data Collection

Between 2006 and 2016, paper headache questionnaires were sent to all ESA and NASA astronauts who had flown on space missions. All astronauts who agreed to participate in the retrospective study provided written informed consent. Completed questionnaires were returned to the study team through the NASA/ESA. All questionnaires received were used for analysis. One astronaut participated in both the prospective and retrospective studies.

### Outcomes

The primary outcome was the occurrence of headache episodes and accompanying symptoms. Headache episodes were defined as periods of headache, preceded and followed by headache-free intervals of at least 4 hours, and classified according to the ICHD-3 criteria.^5^ Headache severity was subjectively rated using an 11-point numeric rating scale (0 = no headache; 10 = worst headache imaginable [nondefined]). Preflight headaches were rated as mild, moderate, or severe. Other headache characteristics, associated symptoms, and use of medication were scored in a dichotomous manner. Used medications were specified. For assessing the short-term effects vs long-term effects of microgravity in the prospective study, we compared the headache episodes from week 1 with later headache episodes. These later episodes were further categorized as within or after the first 13 weeks of mission (halfway mission duration).

In addition to the clinical data, the following ambient data within the ISS were collected on a minute-to-minute basis during the prospective study: levels of carbon dioxide (% relative to total pressure) and oxygen (% relative to total pressure), partial pressure (carbon dioxide; oxygen; millibar), temperature (degrees Centigrade), and relative humidity (percentage). Data were collected using standard NASA/ESA methods and transferred to the study team through the Danish Aerospace Company.

### Statistical Analysis

Analysis of headache data in both studies was performed using a descriptive clinical approach, using chi square tests, McNemar tests, or Fisher exact tests, where appropriate. In the prospective data analysis, data from the 1 participant in short-haul flight were not included because the duration of the space flight was only 10 days. An intraindividual prediction logistic regression analysis was used to determine the relationship between the occurrence of headache during the week and average CO_2_ level (M.J.L.P.). Mission time was used as a within-subject repeated factor and age at launch as covariate. For the analysis of ambient data in relation to occurrence of headaches, we used headache and ambient data from week 3 until the end of a mission. Ambient data were preprocessed: because the diary entries were based on the previous week, average CO_2_ (and O_2_, humidity, temperature, and pressure) levels were calculated over the 7 days before diary entry dates. For some weeks, these measurements were not available and were therefore set as missing in the analyses. Analyses were performed in SPSS 23.0.0 (SPSS Inc., IBM, Armonk, NY), except for the ambient data analysis using MATLAB R2013a (Mathworks, Inc., Natick, MA).

### Standard Protocol Approvals, Registrations, and Patient Consents

The study protocol (Pro 0695) was approved by the ESA Medical Board, NASA Johnson Space Center Committee for the Protection of Human Subjects, later by Johnson Space Center Institutional Review Board, JAXA Institutional Review Board for Human Research, and after that by the ISS Human Research Multilateral Review Board. All astronauts were briefed on the study protocol at the Informed Consent Briefings (A.A.V.) and provided written informed consent before participation in the study.

Both studies were additionally approved by the medical ethics committee of LUMC. All astronauts provided written informed consent before participation. The funders of the studies reported here had no role in study design, data collection, data analysis, data interpretation, or writing of the report. All authors had full access to all the data in this study, and the corresponding author had final responsibility for the decision to submit for publication.

### Data Availability

Data not provided in the article because of space limitations may be shared at the request of qualified investigators for the purpose of replicating procedures and results.

## Results

### Prospective Study at the ISS

#### Participants

**Figure 1:**
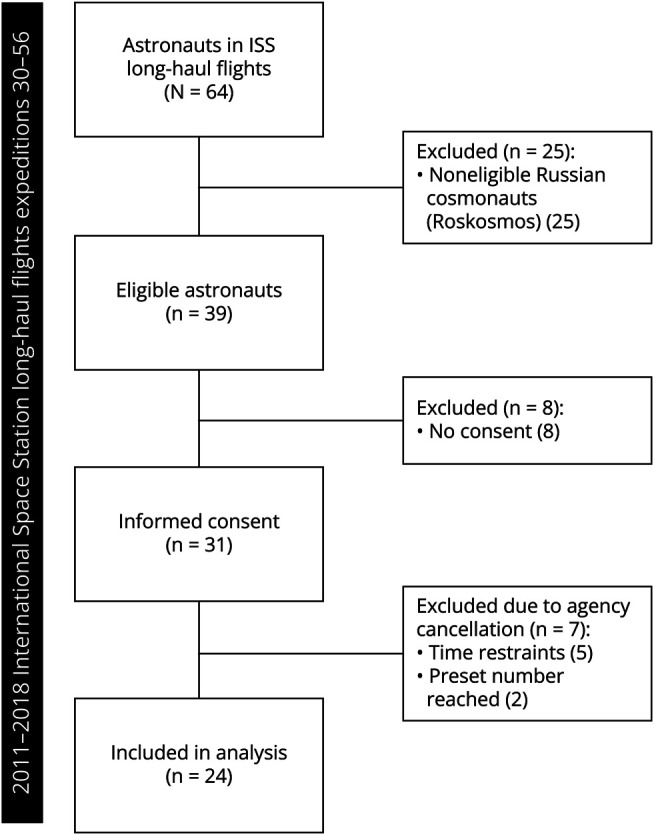
Flowchart of Astronaut Inclusion and Exclusion in the Prospective Study

Of the 39 eligible ESA, NASA, or JAXA crew members who visited the ISS from November 2011 until June 2018, 31 (86.1%) consented before the flight to participate during a total of 21 ISS expeditions (missions 30–56). In 5/31 (19.4%) astronauts from whom baseline data were obtained, in-flight participation was cancelled due to time constraints. Two astronauts did not participate in the in-flight study because the preset number of participants was reached, leaving 24 in-flight participants (Figure 1). All participants completed the study. The study period consisted of 3,596 days of in-flight data collection. In-flight data were missing from 9 measurement points (3 daily questionnaires; 6 weekly questionnaires) due to technical issues, omitting 45/3,596 days (1.3%) from 8 crew members from 7 ISS expeditions from the final analysis. There were no missing data from baseline Earth-based time points.

Of the 24 participants, 23 (95.8%) were male, and the mean age was 46.7 years (SD 6.6; range 36.5–60.3 years). Most of the astronauts (23/24; 95.8%) were part of a long-duration flight (Table 1). Age did not differ between eligible participating (n = 24) and nonparticipating (n = 15) crew members (mean age 46.7 years [SD 6.6] vs 47.4 years [SD 6.6], *p* = 0.76).

**Table 1:** Demographics and Baseline Headache Characteristics in the Prospective Study Population

	Total n = 24 subjects
Demographics	
Age, y, mean ± SD	46.6 ± 6.5
Gender, male, n (%)	23 (95.8%)
Space agency, n (%)	
ESA	6 (25.0%)
NASA	13 (54.2%)
JAXA	4 (16.7%)
CSA	1 (4.2%)
Historical headache data	
Ever headache interfering with daily activities, n (%)	9 (37.5%)
Interfering headache in past 3 mo	3 (12.5%)
Headache frequency in past, n (%)	
Never	9 (37.5%)
1/mo–1/y	13 (54.2%)
1/wk–1/mo	2 (8.3%)
Headache severity untreated headaches (NRS score), mean ± SD	3.1 ± 2.0
Headache severity treated headaches (NRS score), mean ± SD	7.0 ± 2.3
Headache interfering with daily activities in past 12 mo, n (%)	3 (12.5%)
Ever had a headache preceded by visual symptoms, n (%)	2 (8.3%)
Ever diagnosed with migraine by physician, n (%)	0 (0%)

Abbreviations: CSA = Canadian Space Agency; ESA = European Space Agency; JAXA = Japanese Aerospace Exploration Agency; NASA = National Aeronautics and Space Administration; NRS = Numeric Rating Scale (range 0 [no headache] to 10 [worst headache imaginable]).

#### Headache Episodes

The preflight headache history is summarized in Table 1: 9/24 (37.5%) astronauts reported never having any headaches, and 3/24 (12.5%) had had a headache interfering with daily activities in the past 12 months. None of them had a history of recurrent headaches or had ever been diagnosed with migraine.

As specified in the Methods section, for assessing the short-term effects vs long-term effects of microgravity, we compared the occurrence and symptomatology of headache episodes in the first week of the space flight with those at later stages. The later headache episodes were further divided into those occurring in the first half of the space mission (excluding the first week) and those occurring in the second half.

**Figure 2:**
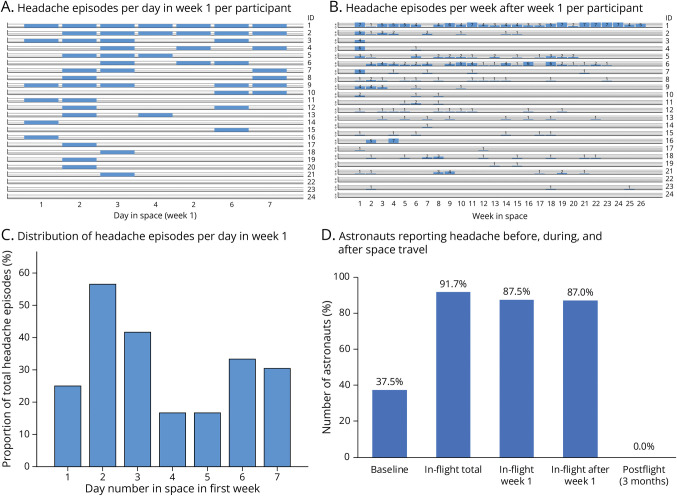
Occurrence of Headache Episodes During Space Flight in Individual Astronauts Data from 1 participant in short-term flight (n = 10 days) are included in the first-week data analysis and graph (A) but are excluded in the later week analyses and graph (B). In Figure 2A, blue bars indicate occurrence of a headache episode on a specific day per individual astronauts, with every row indicating an individual astronaut (ID). Astronauts are ranked in order descending from highest total number of headache episodes in the first week. In Figure 2B, blue bars indicate the number of headache episodes per week in individual astronauts, with every row indicating an individual astronaut (ID). Numbers indicate number of headache episodes per week. Astronauts are ranked in the same order as in Figure 2A (based on descending total number of headache episodes in week 1). ID20 is not included in this analysis because this astronaut participated only in a short-term space flight. Figure 2C depicts the distribution of all first-week headache episodes over the individual days in the first week. Figure 2D During the entire space flight, the proportion of astronauts with headache episodes was higher when compared with that during preflight on-Earth (*p* = 0.0005; McNemar test), without differences between the first and later weeks (*p* = 1.00; McNemar test).

The occurrence and characteristics of the headaches during space flight and the accompanying symptoms are summarized in Table 2. In total, 378 headache episodes were reported in-flight by 22/24 (91.7%) astronauts (vs 9/24 [37.5%] preflight; *p* = 0.0005). The median individual headache frequency was 9 (range 1–128), with >50 episodes reported by 2/22 (9.0%) astronauts and <10 episodes reported by 11/22 (50.0%). Of the 22 astronauts who reported ≥1 headache episode, 2 (9.0%) reported headaches only in the first week and 9/22 (40.9%) only in the first 13 weeks of the mission (first week included). Headaches never occurred only in the second half (weeks 14–26) of the mission. Thus, if astronauts had headaches in the second half of the flight, they also had had headaches in the first half. In 8/22 (36.4%) astronauts, headaches only occurred from week 2 onward (7/8 had ≥50% of headaches in weeks 2–13 and 4/8 had ≤4 headache episodes). Occurrence of headache episodes is graphically depicted in Figure 2, A and B.

**Table 2:** Characteristics of Space Headache Episodes During First and Later Weeks of Space Travel in the Prospective Study

Item	Total (N = 197)	Week 1 (N = 51)	After week 1 (N = 146)	*p* Value Week 1 vs > after week 1
Proportion of astronauts with headache, n (%)	22/24 (91.7)	21/24 (87.5)	20/23 (87.0)	1.00
Unilateral, n (%)	30/195 (15.4)	7/51 (13.7)	23/144 (16.0)	0.82
Severity				0.33
Mild	124/193 (64.2)	36/51 (70.6)	88/142 (62.0)	
Moderate	57/193 (29.5)	11/51 (21.6)	46/142 (32.4)	
Severe	12/193 (6.2)	4/51 (7.8)	8/142 (5.6)	
Increase by exertion, n (%)	42/196 (21.4)	13/51 (25.5)	29/145 (20.0)	0.43
Increase by stress, n (%)^a^	56/196 (28.6)	6/51 (11.8)	50/145 (34.5)	0.002
Character, n (%)^a^				
Pounding	20/194 (10.3)	2/50 (4.0)	18/144 (12.5)	0.11
Pulsating	6/195 (3.1)	0/50 (0)	6/145 (4.1)	0.34
Stabbing	17/195 (8.7)	3/50 (6.0)	14/145 (9.7)	0.57
Pressing	78/195 (40.0)	20/50 (40.0)	58/145 (40.0)	1.00
Exploding	7/195 (3.6)	4/50 (8.0)	3/145 (2.1)	0.07
Monotone	129/195 (66.2)	35/50 (70.0)	94/145 (64.8)	0.60
Heavy feeling	29/193 (14.9)	2/50 (4.0)	27/145 (18.6)	0.01
Headache duration, n (%)				0.001
<1 h	44/178 (24.7)	8/43 (18.6)	36/135 (26.7)	
1–4 h	63/178 (35.4)	7/43 (16.3)	56/135 (41.5)	
4–8 h	32/178 (18.0)	12/43 (27.9)	20/135 (14.8)	
8–12 h	13/178 (7.3)	3/43 (7.0)	10/135 (7.4)	
12–24 h	23/178 (14.0)	13/43 (30.2)	12/135 (8.9)	
Invariable	1/178 (0.5)	0/43 (0.0)	1/135 (0.7)	
Duration, h, mean ± SD	4.8 ± 5.5			
Accompanying symptoms, n (%)				
Nausea^c^	19/196 (9.7)	9/51 (17.6)	10/145 (6.9)	0.05
Vomiting	6/196 (3.1)	5/51 (9.8)	1/145 (0.7)	0.005
Vertigo	3/196 (1.5)	2/51 (3.9)	1/145 (0.7)	0.17
Photophobia	9/196 (4.6)	3/51 (5.9)	6/145 (4.1)	0.70
Phonophobia	9/196 (4.6)	2/51 (3.9)	7/145 (4.8)	1.00
Osmophobia	2/196 (1.0)	0/51 (0)	2/145 (1.4)	1.00
Sleeplessness	38/196 (19.4)	10/51 (19.6)	28/145 (19.3)	1.00
Nasal congestion	70/196 (35.7)	27/51 (52.9)	43/145 (29.7)	0.004
Visual disturbances	3/196 (1.5)	1/51 (2.0)	2/145 (1.4)	1.00
Facial edema	23/196 (11.7)	21/51 (41.2)	2/145 (1.4)	<0.001
No. of symptoms				
Mean ± SD	0.9 ± 1.4	1.6 ± 1.8	0.70 ± 1.1	0.001
Median, range	0, 0–7	1, 0–7	0, 0–6	0.003
Treatment				
Any treatment, n (%)	134/192 (69.8)	32/50 (64.0)	102/142 (71.8)	0.37
No. of treatments, n (%)				0.76
0	58/192 (30.2)	18/50 (36.0)	40/142 (28.2)	
1	74/192 (38.5)	17/50 (34.0)	57/142 (40.1)	
2	45/192 (23.4)	11/50 (22.0)	34/142 (23.9)	
3	15/192 (7.8)	4/50 (8.0)	11/142 (7.7)	
Use of medication, n (%)	87/196 (55.6)	22/51 (43.1)	65/145 (44.8)	0.87
Promethazine	4/87 (4.6)	4/27 (14.8)	0/58 (0)	0.003
NSAID	67/87 (77.0)	25/27 (92.6)	42/58 (72.4)	0.03
Sudafed	2/87 (2.3)	1/27 (3.7)	1/58 (1.7)	0.58
Aspirin	8/87 (9.2)	2/27 (7.4)	6/58 (10.3)	0.67
Antihistamine	5/87 (5.7)	2/27 (7.4)	3/58 (5.2)	0.68
Dexmethylphenidate	1/87 (1.1)	1/27 (3.7)	0/58 (0)	0.14
Acetaminophen	14/87 (16.1)	3/27 (11.1)	11/58 (19.0)	0.36
Scopolamine	4/87 (4.7)	4/27 (14.8)	0/58 (0)	0.003
Dexamfetamine	3/87 (3.4)	3/27 (11.1)	0/58 (0)	0.01
Effect of medication, n (%)	73/84 (86.9)	18/21 (85.7)	55/63 (87.3)	1.00
Alternative treatment^b^	79/192 (41.1)	14/50 (28.0)	65/142 (45.8)	0.03
Use other medication	44/84 (52.4)	15/51 (29.4)	29/144 (20.1)	0.18
Second treatment, n (%)				
Alternative treatment	38/86 (43.7)	9/22 (40.9)	14/65 (21.5)	0.10
Use other medication 2	23/87 (26.4)	10/22 (45.5)	28/64 (43.1)	1.00
Alternative treatment, n (%)				
Eating	12/38 (31.6)	3/12 (25.0)	9/27 (36.0)	0.50
Sleeping	16/38 (42.1)	3/12 (25.0)	6/25 (24.0)	0.95
Hydration	15/38 (39.5)	7/12 (58.3)	8/25 (32.0)	0.13
Exercise	8/38 (21.1)	1/12 (8.3)	7/25 (28.0)	0.17
Caffeine beverages	9/38 (23.7)	3/12 (25.0)	6/25 (24.0)	0.95

Abbreviation: NSAID = nonsteroidal anti-inflammatory drug.

In case of small numbers (n per cell <10), the Fisher exact test and nonparametric tests were used appropriately instead of a χ^2^ test and Student *t* test.

a Answer options were not mutually exclusive.

b Including rehydration, sleep, and intake of coffee.

c In the questionnaires, “stress” was not further specified.

Detailed information about 197/378 (52%) episodes was available for analysis (of which for 189, sufficient data were available for classification of headache subtypes, although not all details were available for all episodes). Overall, 170/189 (89.9%) headache episodes phenotypically fulfilled the criteria for tension-type headache (TTH) or probable TTH and 19/189 (10.1%) for migraine or probable migraine. Overall, the headaches were mostly mild (124/193; 64.2%), ≤4 hours (107/178; 60.1%), and of monotonous character (129/195; 66.2%). Most common accompanying symptoms were nasal congestion (70/196; 35.7%) and sleeplessness (38/196; 19.4%). The mean number of accompanying symptoms per headache episode was 0.9 ± 1.4.

In the first week, 21/24 (87.5%) participants had ≥1 headache episode (median 2; interquartile range [IQR] 2; range 1–6 episodes), and there were 51 episodes in total. Headaches were most often mild (36/51; 70.6%) and of a monotonous (35/50; 70.0%) quality. In 20/50 (40.0%), they were pressing and in 13/51 (25.5%) aggravated by exertion. Nasal congestion (27/51; 52.9%) and facial edema (21/51; 41.2%) were the most prevalent accompanying symptoms. Nausea (17.6%) and vomiting (9.8%) were also reported. Of the 51 episodes, 12 (23.5%) fulfilled the criteria for migraine or probable migraine in 3 persons and 39 (86.5%) for TTH or probable TTH in 21 persons (100%). Headache most frequently occurred at the second day in space (56.5%; *p* = 0.02; Figure 2C), after which the prevalence declined.

In the following weeks, 20/23 (87.0%; 1 astronaut participated only in a short-term flight) ISS crew members reported a total of 327 in-flight headache episodes (median 8.5; IQR 9.5; range 1–123). Data from 146/327 (45%) episodes were available for analysis, but not all details were available for all episodes. The headaches were most often mild (88/142; 62.0%), with an increasing severity with experienced stress (50/145; 34.5%), and had a pressing (58/145; 40.0%) and monotonous (94/145; 64.8%) quality. Nasal congestion (43/145; 29.7%) and sleeplessness (28/145; 19.3%) were the most commonly associated symptoms. Of the 138 episodes with sufficiently available details, 131 (94.9%) fulfilled ICHD-3 criteria for TTH or probable TTH in 20 persons and 7 (5.1%) for migraine or probable migraine in 5 persons.

**Figure 3:**
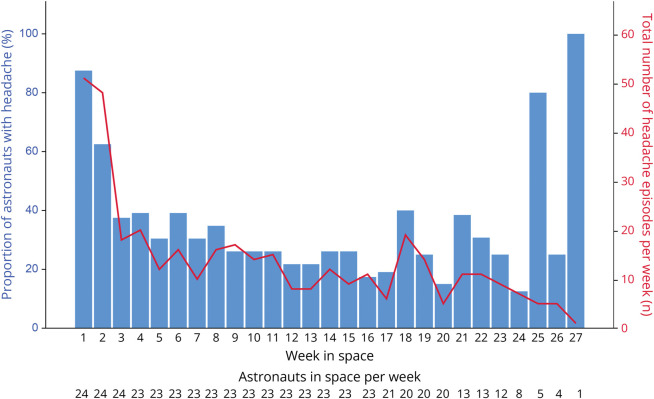
Occurrence of Headache Episodes During Long-Term Space Travel in 24 Astronauts Declines After the First Week in Space After the first week in space, both the proportion of astronauts reporting headache episodes (left y-axis) and the mean number of headache episodes reported per week/participating astronaut (right y-axis) decrease and remain more or less stable over time. As the number of astronauts gets very small after week 24, data points have larger standard errors (which have been omitted for reasons of clarity of the graph).

There was no difference in the proportion of astronauts with a headache episode in the first week vs overall later weeks: 21/24 (87.5%) vs 20/23 (87.0%; *p* = 1.00) (Figure 2D). Episodes in the later weeks more often were phenotypically TTH than those in the first week: 131/138 (94.9%) vs 39/51 (76.5%) (*p* = 0.0002). The later headache episodes more often were of a heavy feeling (18.6% vs 4.0%; *p* = 0.01), shorter duration (*p* = 0.001), and increasing severity with increasing stress (34.5% vs 11.8%; *p* = 0.002). Later episodes had less associated symptoms (0.7 ± 1.1 vs 1.6 ± 1.8; *p* = 0.002), for example, nausea, vomiting, nasal congestion, and facial edema (all *p* < 0.05) (see eTable 1, links.lww.com/WNL/D465). Headache occurrence decreased after the first week in space (*p* = 0.05) and remained stable afterward (*p* = 0.49; trend analysis; Figure 3). In the 3 months after return to the Earth, none of the astronauts reported any headache episodes.

#### Treatment and Countermeasures

In 134/192 (69.8%) episodes, at least 1 form of treatment was used to treat the headache (without differences between week 1 and later): use of acute antiheadache medication (87/196; 55.6%), other medication (44/196; 22.4%), or alternative treatment (coffee, rehydration, and exercise: 79/192; 41.1%). Sixteen of 21 (76.2%) ISS crew members with headaches reported taking acute antiheadache medication in flight with good efficacy in 73/84 (86.9%) episodes. NSAIDs (67/87; 77.0%) and acetaminophen (14/87; 16.1%) were overall the most frequently taken acute antiheadache drugs on ISS expeditions. In the first week, promethazine, scopolamine, and dexamphetamine (nonspecific, general space travel medication available onboard the ISS) were taken more often than later in the following weeks. In 60/192 (31.2%) episodes, more than 1 treatment was used. Reported additional measures and countermeasures were most often sleeping (16/38; 42.1%) and hydration (15/38; 39.5%), Table 2.

#### Headache and Environmental Factors in the ISS

**Figure 4:**
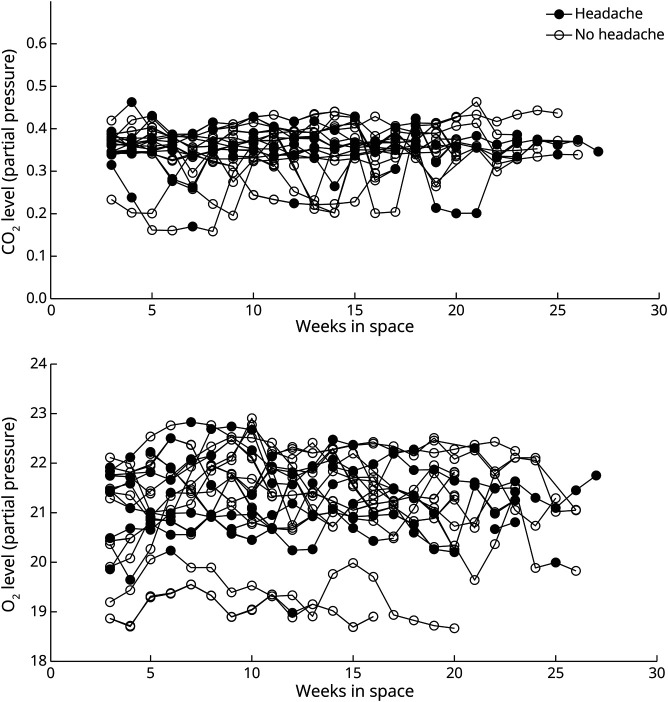
Graph Showing There Was No Relation Between Partial CO_2_ and Partial O_2_ Levels and Weekly Headache Incidence in Individual Healthy Astronauts During Long-Term Stay at the ISS Every line reflects data from individual astronaut during space stay, in both figures.

Weekly mean CO_2_ levels, O_2_ levels, humidity, temperature, or pressure did not differ from weeks in which an individual astronaut experienced headache vs weeks wherein the astronaut did not experience a headache (eTable 2, links.lww.com/WNL/D465). In addition, there was no correlation between mean CO_2_ levels,O_2_ levels, and individually reported headache episodes (Figure 4).

### Retrospective Study

#### Participants

In 2016, retrospective questionnaires were sent out through the agencies in 1 batch (with 1 reminder) to all astronauts who had flown between 2006 and 2016, except for the 17 astronauts who had already been published before.^4^ Of the 67 eligible astronauts who were invited through the space agencies, 42 (63%) agreed to participate and returned the questionnaire by July 2019. Of these, 38/42 (90.5%) were male. The mean age was 43.5 ± 7.2 years, and the mean mission duration was 134.6 ± 83.8 days (Table 3). Data from 17 astronauts have been previously reported.^4^

**Table 3:** Baseline Characteristics and Headache Occurrence Among Astronauts During Space Flight, Based on a Retrospective Questionnaire Study

Variable	Total N = 42 subjects/N = 42 flights
Demographics	
Gender, male, n (%)	38/42 (90.5)
Age, y, mean ± SD	43.5 ± 7.2
Flight data	
Duration, d, mean ± SD	134.6 ± 83.8
Launch vehicle, n (%)	
Soyuz	31 (73.8)
Space shuttle	11 (26.2)
Space station, n (%)	
Mir	6 (14.3)
ISS	35 (83.3)
Salyut	1 (2.8)
Headache data, n (%)	
Headache any time in orbit	23/42 (54.8)
Headache at launch	9/42 (21.4)
Headache in first week	
Headache after first week	
Headache during stays	18/42 (42.9)
Headache at EVA	1/42 (2.4)
Headache during landing	1/42 (2.4)
Ever headache on the Earth^a^	13/42 (30.9)
Medication data	
Took any medication, n (%)	13/23 (56.5)
Medication	
Acetaminophen	1/23 (4.3)
NSAID	4/23 (17.4)
Aspirin	1/23 (4.3)
Medication effect, n (%)	11/13 (84.6)
Alternative treatment, n (%)	4/23 (17.4)
Water/coffee/caffeinated tea	1/23 (4.3)
Rest/sleep	1/23 (4.3)
Shoulder neck massage	1/23 (4.3)
Hydrocortisone	1/23 (4.3)

Abbreviation: EVA = extra vehicular activity; NSAID = nonsteroidal anti-inflammatory drug.

a Before or after flight headache on the Earth (2016)/before flight headache on the Earth (2008).

#### Headache Occurrence and Characteristics

A total of 23/42 (54.8%) astronauts reported 33 headache episodes at any time during in-orbit space flight. Headaches were mostly of mild-to-moderate severity (31/33; 93.9%) and monotonous or heavy in quality (32/33; 97.0%) with transient vertigo, nausea, and photophobia during the first week. Nasal congestion (6/33; 12.8%) was the most commonly reported associated symptom (eTable 1, links.lww.com/WNL/D465). Of all 33 episodes, 2 (6.1%) were classified as migraine or probable migraine, both occurring in the first week of mission.

## Discussion

We investigated the occurrence, timing, and characteristics of headache and possible associated symptoms during long-term space-flights in 66 super healthy astronauts, who did not have regular headaches previously in the Earth. Prospective data were collected in 24 astronauts, and retrospective data from 42 astronauts were used to confirm the prospective findings. We found that episodes of headache were provoked throughout the space flight, but with different symptomatology and incidence. Whereas a migraine phenotype with associated nausea, vomiting, facial edema, and nasal congestion was more prevalent in the first week, a TTH phenotype predominated in the later phases of microgravity. Occurrence of headache episodes declined and duration of headache episodes shortened with longer stay in microgravity. There was no correlation of space headache with changes in ambient parameters in the space missile or station. After returning to the Earth, none of the astronauts reported any headaches again.

We can only speculate about the etiology of space headache. Two different mechanisms might play a role. In the first week, adaptation to weightlessness occurs with a prominent role for the vestibular system. This phenomenon is described by the vestibular sensory conflict hypothesis.^7^ Loss of tilt-related otolith signals in the absence of normal gravity is suggested to cause a conflict between actual and anticipated signals from sensory organs subserving spatial orientation. Sensory conflict is considered the primary cause of space motion sickness of which headache is the most frequently reported symptom.^7,9,10^ Results from our study support this assumption: we found that headache in combination with transient nausea, vomiting, and vertigo almost exclusively occurred during the first week in flight.

During longer stay in space, the cephalad redistribution of body fluids might result in intracranial and extracranial fluid shifts, increasing the intracranial pressure and triggering headaches (eFigure 1, links.lww.com/WNL/D465). This hypothesis is appealing because cephalad fluid shifts of >100 cc are not very likely to be without functional consequences.^11,12^ We showed that the clinical picture of space headache after the first week in microgravity resembles the milder forms of on-Earth headache due to intracranial hypertension, in which a phenotypically TTH is the main and most common symptom.^13^ Simultaneously, we have observed remarkable interindividual differences in headache patterns, suggesting not-yet identified susceptibility factors must play an additional role.

Of interest, the most commonly reported associated symptom was nasal congestion, which is caused by swelling of the nasal mucosa due to this increase in extracerebral fluid volume. Papilledema and progressive visual loss are linked to on-Earth intracranial hypertension.^14,15^ A recent study reported additional evidence for this hypothesis: visual symptoms and/or ocular abnormalities (optic disc swelling, globe flattening, choroidal folds, and hyperopic shifts in refraction) occur in 29%–60% of astronauts post flight. These are interpreted as signs of the visual impairment intracranial pressure (VIIP) syndrome, postulated to be the result of increased intraocular pressure due to microgravity.^16^ In addition, MR studies of the brains of astronauts showed a reversible increase in ventricular volumes post flight,^1,17^ and there is evidence for persisting CSF circulation disturbances many months after return to the Earth.^18^ This might explain in-flight space headache and ocular/visual abnormalities after long-duration spaceflight.

In-flight gravitational countermeasures such as exercise and transient enhanced artificial gravity aim to limit the effect of microgravity-induced physiologic changes.^12^ Previously, we reported that headache intensity and accompanying symptoms decreased with countermeasures in an on-Earth simulation model.^19^ High-intensity exercise is suggested to decrease intracranial pressure^12^ and could thereby also limit the burden of microgravity-induced headaches. Although our study was not primarily aimed at assessing the effects of countermeasures, astronauts did often report headache relief after sleep, exercise, rehydration, and coffee intake. Intake of analgesics, mainly NSAIDs, was also reported as an effective relief in many headache episodes. Previously, increased onboard CO_2_ levels had been related to the occurrence of headache episodes.^20^ We could not confirm this in our prospective study.

Few studies investigated the effect of microgravity on pain. Most addressed back, leg, or abdominal pain^21,22^ with little detail for headache characteristics.^21^ Although headache has been sporadically reported as a common symptom in space, detailed analysis of the clinical characteristics was lacking.^3,23,24^ Earlier publications from our group, using data from retrospective and simulation studies, are well in line with the results from this study.^4,19,25^ Space headache has now been recognized as a new entity in the ICHD.^5^

Our study has several strengths. We were able to comprehensively and prospectively monitor headache and associated symptoms during an actual long-term stay in space in 66 astronauts. Because of logistic and financial reasons, such data are usually collected during on-Earth studies with simulated microgravity^19,21^ or directly after return from Space.^22^ Only sleep quality in space has been studied in a similar prospective manner.^2^ With our prospective findings, we confirmed previous retrospective in-flight^4^ and simulated microgravity data.^19,25^ A total of 66 astronauts is substantial in space medicine research.^26^ Unfortunately, because there were only 5 female astronauts in our study, we cannot reliably extend our findings to women, in whom headache is more common on the Earth.^27^ Missing data were very few, and the findings in the retrospective and prospective parts of the study were consistent and complimentary, increasing the validity of our observations.^26^

In conclusion, space flights provoked headaches in super healthy people who did not have regular headaches before and after the space mission.^28,29^ space headaches occurred during the whole mission, but most often in the first week and were then often of the migraine type. Space headaches occurring in later stages of the flights were less frequent and mainly of a TTH, suggesting a different underlying mechanism. Further research is needed to unravel the underlying mechanisms of space headache and translate them to headaches occurring on the Earth. In addition, more effective therapies need to be developed to combat space headaches because for many astronauts, this a major recurring disabling problem during space flights.

## Appendix Authors

**Table:**

Name	Location	Contribution
Willebrordus P.J. van Oosterhout, MD, PhD	Department of Neurology, Leiden University Medical Center; Department of Neurology, Zaans Medical Center, Zaandam, the Netherlands	Drafting/revision of the article for content, including medical writing for content; major role in the acquisition of data; study concept or design; and analysis or interpretation of data
Matthijs J.L. Perenboom, MSc	Department of Neurology, Leiden University Medical Center, the Netherlands	Drafting/revision of the article for content, including medical writing for content; analysis or interpretation of data
Gisela M. Terwindt, MD, PhD	Department of Neurology, Leiden University Medical Center, the Netherlands	Drafting/revision of the article for content, including medical writing for content; study concept or design; and analysis or interpretation of data
Michel D. Ferrari, MD, PhD	Department of Neurology, Leiden University Medical Center, the Netherlands	Drafting/revision of the article for content, including medical writing for content; study concept or design; and analysis or interpretation of data
Alla A. Vein, MD, PhD	Department of Neurology, Leiden University Medical Center, the Netherlands	Drafting/revision of the article for content, including medical writing for content; study concept or design; analysis or interpretation of data; and additional contributions: principal investigator of the ESA experiment Pro 0695 “Space Headache: incidence and characteristics”

## Glossary

CSA: Canadian Space Agency
ESA: European Space Agency
ICHD-2: International Classification of Headache Disorders, second edition
ICHD-3: International Classification of Headache Disorders, third edition
IQR: interquartile range
ISS: International Space Station
JAXA: Japan Aerospace Exploration Agency
LUMC: Leiden University Medical Center
NASA: National Aeronautics and Space Administration
TTH: tension-type headache
